# Effect of HPV Adult Vaccination on Serum Anti-Müllerian Hormone Levels: Paired Measurements in a Retrospective Cohort

**DOI:** 10.3390/vaccines14030233

**Published:** 2026-03-03

**Authors:** Ali Can Gunes, Muhammed Onur Atakul, Utku Akgor, Gonca Ozten Dere, Murat Cengiz, Haticegul Tuncer, Betul Gungor Serin, Mehmet Kabacam, Hakan Aydinli, Murat Gultekin

**Affiliations:** 1 Department of Obstetrics and Gynecology, Faculty of Medicine, Hacettepe University, Ankara 06100, Turkey; onuratakul@hacettepe.edu.tr (M.O.A.); goncaozten@hacettepe.edu.tr (G.O.D.); haticegul.tuncer@hacettepe.edu.tr (H.T.); betulgungor@hacettepe.edu.tr (B.G.S.); 2 Department of Obstetrics and Gynecology, Division of Gynecologic Oncology, Faculty of Medicine, Hacettepe University, Ankara 06100, Turkey; utkuakgor@hacettepe.edu.tr (U.A.); muratcengiz@hacettepe.edu.tr (M.C.); gultekin@hacettepe.edu.tr (M.G.); 3 Faculty of Medicine, Hacettepe University, Ankara 06230, Turkey; mehmetkabacam@hacettepe.edu.tr (M.K.); hakanaydinli@hacettepe.edu.tr (H.A.)

**Keywords:** human papillomavirus, HPV vaccination, anti-Mullerian hormone, ovarian reserve, vaccine safety

## Abstract

Background: Concerns that human papillomavirus (HPV) vaccination may adversely affect ovarian reserve contribute to vaccine hesitancy, yet longitudinal data with paired anti-Müllerian hormone (AMH) measurements are limited. We evaluated whether HPV vaccination was associated with short-term changes in AMH compared with an unvaccinated control group. Methods: In this retrospective cohort, women aged 18–45 years who completed a three-dose 9-valent HPV vaccination (Gardasil 9®, Merck Sharp & Dohme LLC, West Point/Pennsylvania/USA) schedule and had AMH measured before dose 1 and after dose 3 were compared with unvaccinated controls who had two AMH measurements during routine gynecologic evaluation. AMH change was summarized as absolute change (ΔAMH), percent change, and log change. To compare rates of AMH change while accounting for heterogeneous follow-up and confounding, AMH was analyzed on the natural log scale using a linear mixed-effects model with a random intercept for participant and fixed effects for time (years), group, and a time × group interaction, adjusted for age, current smoking, gravidity, and parity. Annual percent change was derived from model coefficients. Prespecified sensitivity analyses repeated the primary model under follow-up restrictions and after restricting baseline AMH to 1.0–5.0 ng/mL. Results: The cohort included 158 vaccinated and 106 control women. Baseline AMH was similar between groups (median 1.88 vs. 1.94 ng/mL), while the follow-up interval was shorter in vaccinated women (6.7 vs. 8.9 months). Unadjusted AMH decline was smaller in vaccinated women (median ΔAMH −0.13 vs. −0.27 ng/mL; *p* = 0.015; median percent change −10.9% vs. −20.6%; *p* = 0.006). In the adjusted mixed-effects model, controls showed an estimated AMH decline of −27.6% per year (95% CI −35.5% to −18.7%; *p* < 0.001). The time × group interaction was positive (β = 0.170, 95% CI 0.027 to 0.312; *p* = 0.020), corresponding to a slope ratio of 1.185 (95% CI 1.02–1.366) and an implied annual change of −14.2% per year (95% CI −21.0% to −6.7%) in vaccinated women. Results were broadly consistent in follow-up-restricted sensitivity analyses; however, in the baseline AMH 1.0–5.0 ng/mL restricted cohort (vaccinated n = 82, control n = 67), the interaction was attenuated and not statistically significant (β = 0.082, *p* = 0.237). Conclusions: In this retrospective cohort with paired AMH measurements, HPV vaccination was not associated with evidence of clinically meaningful short-term impairment in ovarian reserve as assessed by AMH. Observed differences in AMH alterations were modest and should be interpreted cautiously, given residual confounding, measurement variability, and reduced precision in restricted-cohort analyses.

## 1. Introduction

Human papillomavirus (HPV) infection is among the most common sexually transmitted viral infections worldwide and is a major cause of cervical cancer; consequently, almost all cases of cervical cancer are attributable to persistent infection with oncogenic HPV type [1,2,3]. Beyond the cervix, persistent infection with high-risk HPV types is also causally linked to a substantial proportion of other anogenital and oropharyngeal malignancies, including anal, vulvar, vaginal, penile, and oropharyngeal cancers [4,5,6]. From an epidemiologic perspective, HPV is highly prevalent in reproductive-age populations [7], and the global burden of HPV-attributable disease remains considerable, with geographic variation in incidence and mortality that reflects differences in screening and vaccine uptake [8,9]. Prophylactic HPV vaccination is therefore a cornerstone of cervical cancer prevention and has demonstrated substantial population-level benefits, including marked reductions in HPV infections, high-grade cervical lesions, and cervical cancer incidence in settings with high vaccine uptake [10,11,12]. Despite this well-established public health value, vaccine hesitancy persists in many countries, partly driven by concerns about potential effects on reproductive health [13,14]. During the study period in Turkey, prophylactic HPV vaccination was administered according to age-based recommendations; individuals aged ≥15 years typically received a three-dose schedule (0, 1–2, and 6 months), whereas younger adolescents were eligible for a two-dose schedule. In our cohort, vaccination exposure corresponded to the 9-valent HPV vaccine (Gardasil 9®, Merck Sharp & Dohme LLC, West Point/Pennsylvania/USA) given as a three-dose series, reflecting real-world practice in adult women.

Ovarian reserve is a key determinant of female reproductive potential and is commonly assessed using serum anti-Müllerian hormone (AMH), produced by granulosa cells of small growing follicles and strongly correlated with the number of antral follicles [15,16]. AMH is widely used in reproductive medicine for counseling and treatment planning; however, AMH values show within-person biological variation and are also influenced by analytical (assay-related) variability [17,18]. Accordingly, short-term changes in AMH may reflect aging, intercurrent factors, and measurement timing rather than a causal effect of an exposure [17,18].

Concerns regarding a possible association between HPV vaccination and ovarian function have been raised in public discourse and initially amplified by case reports of premature ovarian insufficiency (POI) temporally following HPV vaccination [19,20]. In contrast, large-scale epidemiologic analyses have not supported an increased risk of POI following HPV vaccination, and systematic reviews have generally provided reassuring evidence regarding ovarian safety [14,20,21]. Moreover, many studies assessing fertility-related outcomes after vaccination are cross-sectional, rely on self-reported vaccination status, or lack pre-exposure measurements, limiting causal interpretation [20,21]. Longitudinal assessments with paired AMH measurements and appropriate handling of follow-up time are therefore valuable to clarify whether HPV vaccination is associated with measurable short-term changes in AMH.

In this context, we conducted a retrospective cohort study using electronic hospital records to compare AMH alterations in women who completed a three-dose HPV vaccination series and a contemporaneous control group. Using paired baseline and follow-up AMH measurements and modeling change over time while accounting for potential confounders, our objective was to evaluate whether HPV vaccination is associated with short-term changes in AMH.

## 2. Materials and Methods

This retrospective cohort study was conducted at the Department of Obstetrics and Gynecology, Hacettepe University Faculty of Medicine, Ankara, Turkey. Electronic hospital records were reviewed to identify women who received 9-valent HPV vaccination (Gardasil 9®, Merck Sharp & Dohme/MSD) between January 2022 and October 2025. A contemporaneous control group was identified from women aged 18–45 years who attended the general gynecology outpatient clinic for routine gynecologic evaluation during the same period and had AMH measured as part of clinical evaluation.

The vaccinated cohort was eligible if women: (i) received a complete 3-dose HPV vaccination schedule between 2022 and 2025; (ii) had a documented pre-vaccination AMH measurement; (iii) had an AMH measurement obtained after completion of the third dose; (iv) were aged 18–45 years; and (v) had sufficient demographic and clinical data available for analysis.

Women in the control group were eligible if they were aged 18–45 years, attended the general gynecology outpatient clinic for routine care, had AMH measured, and met the same exclusion criteria applied to the vaccinated cohort.

For both cohorts, exclusion criteria were: oophorectomy, ovarian cystectomy with tissue loss or surgery for ovarian torsion; prior chemotherapy, pelvic radiotherapy, or other known gonadotoxic exposures; documented diagnosis of endometrioma/endometriosis; pregnancy at the time of AMH testing or pregnancy occurring between measurements (Beta hCG positivity regardless of the outcome, such as spontaneous abortion or live birth); uncontrolled endocrine conditions or exposures potentially affecting ovarian function (e.g., uncontrolled thyroid dysfunction, hyperprolactinemia, polycystic ovarian syndrome, or hormonal contraceptive use for any reason); and missing or inconsistent electronic records. In the vaccinated cohort, women were additionally excluded if they did not complete all three HPV vaccine doses or lacked either a pre-vaccination or a post-third-dose AMH measurement.

Strict 1:1 matching was not performed because eligibility required paired AMH measurements and prespecified exclusions, which reduced the pool of eligible controls. Therefore, comparability was addressed using covariate-adjusted mixed-effects modeling on log(AMH) with explicit handling of follow-up time (including a time × group interaction), supported by prespecified sensitivity analyses. Between-group balance was additionally summarized using standardized mean differences (SMDs) in Table 1. Residual confounding due to unmeasured covariates cannot be excluded and is acknowledged in the limitations.

Due to non-standardized cycle timing, we did not include cycle-dependent FSH/estradiol measurements, and because AFC is operator-dependent and performed by multiple clinicians, we did not analyze AFC. We therefore focused on AMH as the primary marker for longitudinal comparison in this retrospective real-world cohort. AMH testing in our setting is commonly performed in routine gynecology care (e.g., fertility counseling, ovarian reserve assessment, or routine gynecologic examination) and was not protocolized for this study. 

AMH measurement time points were defined as follows: baseline AMH, the serum AMH measurement obtained prior to any HPV vaccine dose and closest to the date of the first dose; and follow-up AMH, the earliest available AMH measurement performed after completion of the third dose. The primary outcome was the change in AMH between baseline and follow-up measurements.

Baseline demographic and reproductive characteristics included age (years), gravidity, parity, and current smoking status. Body mass index (BMI) was not routinely recorded in our electronic medical records for all patients. BMI values were more likely to be documented when clinicians perceived the patient as markedly underweight or overweight, whereas BMI was frequently missing in patients assessed as having a “normal” habitus. Therefore, BMI could not be used as a covariate without introducing substantial missingness and potential measurement/recording bias. Smoking was analyzed as a binary variable (current smoker: yes/no). In addition, baseline AMH (anti-Müllerian hormone; ng/mL) and the follow-up interval (time between baseline and follow-up AMH measurements, reported in months) were summarized and compared between groups to characterize baseline ovarian reserve and measurement timing (Table 1).

For each participant, AMH change between baseline and follow-up was summarized using multiple metrics: absolute change (ΔAMH = AMH_follow-up – AMH_baseline), percent change (ΔAMH/AMH_baseline × 100), and log change (ln[AMH_follow-up] − ln[AMH_baseline] = ln[follow-up/baseline]) (Table 2).

Continuous variables were presented as median [interquartile range (IQR)] and compared using the Mann–Whitney U test. Categorical variables were presented as n (%) and compared using the chi-square test (or Fisher’s exact test when appropriate). All tests were two-sided, and *p* < 0.05 was considered statistically significant.

To compare longitudinal AMH change between groups while accounting for differences in follow-up time and potential confounding, AMH was analyzed on the natural log scale using a linear mixed-effects model with a random intercept for each participant. Fixed effects included time (years), group (vaccinated vs. control), and a time × group interaction, with additional adjustment for age, current smoking, gravidity, and parity. Results are reported as model coefficients on the log scale; annual percent change was derived as (e^β − 1) × 100, and the slope ratio (vaccinated vs. control) as e^(β_[time × group]). Model fit assumptions were assessed by visual inspection of residual and fitted-value patterns. Statistical analyses were performed using Python 3.11.2 (pandas 2.2.3, SciPy 1.14.1, statsmodels 0.14.3).

Robustness of the primary findings was evaluated in prespecified sensitivity analyses by repeating the primary mixed-effects model after restricting the follow-up interval to ≥3 months and to 3–18 months (Table 4). To minimize the potential influence of extreme baseline ovarian reserve phenotypes, we additionally repeated the primary mixed-effects model after restricting the cohort to participants with baseline AMH between 1.0 and 5.0 ng/mL (Table 4). As an alternative modeling approach, we performed a multivariable linear regression on annualized log change, defined as (ln[AMH_follow-up] − ln[AMH_baseline])/follow-up years, using HC3 robust standard errors and adjusting for baseline log AMH and the same covariates as in the primary model (Table 4).

## 3. Results

A total of 158 women were included in the HPV-vaccinated group and 106 women in the control group. Baseline characteristics are presented in Table 1. Median age was 34.0 [28.0–39.0] years in the vaccinated group and 32.5 [28.2–36.0] years in controls (*p* = 0.067). Gravidity was higher in vaccinated participants (1.0 [0.0–2.0]) than in controls (0.0 [0.0–1.0]; *p* = 0.005), whereas parity was similar (0.0 [0.0–1.0] in both groups; *p* = 0.834). Current smoking was more frequent in the vaccinated group (49/158, 31.0%) compared with controls (17/106, 16.0%; *p* = 0.009). Baseline AMH levels were comparable between groups (1.88 [0.65–3.41] ng/mL vs. 1.94 [0.77–3.20] ng/mL; *p* = 0.879). The follow-up interval was shorter in the vaccinated group (6.7 [4.3–8.9] months) than in controls (8.9 [6.7–11.2] months; *p* < 0.001). These between-group differences—particularly gravidity, smoking, and follow-up interval—were considered potential confounders and were adjusted for in subsequent adjusted analyses of AMH change.

**Table 1:** Baseline characteristics of the cohort.

	Vaccinated (*n* = 158)	Control (*n* = 106)	*p* Value	SMD
Age (years)	34.0 [28.0–39.0]	32.5 [28.2–36.0]	0.067	0.141
Gravidity	1.0 [0.0–2.0]	0.0 [0.0–1.0]	0.005	0.315
Parity	0.0 [0.0–1.0]	0.0 [0.0–1.0]	0.834	−0.032
Current smoker, n (%)	49 (31.0%)	17 (16.0%)	0.009	0.166
Baseline AMH (ng/mL)	1.88 [0.65–3.41]	1.94 [0.77–3.20]	0.879	−0.155
Follow-up interval (months)	6.7 [4.3–8.9]	8.9 [6.7–11.2]	<0.001	0.257

Continuous variables are presented as median [Q1–Q3] and compared using the Mann–Whitney U test. Categorical variables are presented as n (%) and compared using the chi-square test (Fisher’s exact test when appropriate). The follow-up interval represents the time between baseline and follow-up AMH measurements. SMDs were calculated to summarize between-group differences (continuous: mean difference/pooled SD; binary: proportion difference/√(*p*(1−*p*))). AMH, anti-Müllerian hormone (ng/mL). SMD, standardized mean difference. SD, standard deviation.

Among women with paired baseline and follow-up AMH measurements, follow-up AMH was not statistically different between groups (1.85 [0.55–3.21] ng/mL vs. 1.45 [0.58–2.62] ng/mL; *p* = 0.497) (Table 2). Unadjusted AMH decline appeared smaller in the vaccinated group compared with controls: median ΔAMH was −0.13 [−0.62–0.16] ng/mL versus −0.27 [−0.77–0.05] ng/mL (*p* = 0.015), and median percent change was −10.9% [−32.4–17.2] versus −20.6% [−41.1–−7.4] (*p* = 0.006). Similarly, log change was −0.115 [−0.392–0.159] in the vaccinated group compared with −0.230 [−0.530–−0.076] in controls (*p* = 0.006). However, the magnitude of these crude between-group differences appeared modest, with small-to-moderate effect sizes (rank-biserial r ≈ 0.18–0.20 across change metrics).

**Table 2:** Unadjusted comparison of AMH change between groups.

	Vaccinated (*n* = 158)	Control (*n* = 106)	*p* Value
Baseline AMH (ng/mL)	1.88 [0.65–3.41]	1.94 [0.77–3.20]	0.879
Follow-up AMH (ng/mL)	1.85 [0.55–3.21]	1.45 [0.58–2.62]	0.497
Follow-up interval (months)	6.7 [4.3–8.9]	8.9 [6.7–11.2]	<0.001
Absolute change, ΔAMH (post–pre; ng/mL)	−0.13 [−0.62–0.16]	−0.27 [−0.77–−0.05]	0.015
Percent change (%)	−10.9 [−32.4–17.2]	−20.6 [−41.1–−7.4]	0.006
Log change (ln[post]–ln[pre])	−0.115 [−0.392–0.159]	−0.230 [−0.530–−0.076]	0.006

Values are median [Q1–Q3]. ΔAMH was calculated as follow-up AMH minus baseline AMH. Percent change = (ΔAMH/baseline AMH) × 100. Log change = ln(follow-up AMH) − ln(baseline AMH) = ln(follow-up/baseline). Between-group comparisons used Mann–Whitney U test. AMH, anti-Müllerian hormone (ng/mL).

**Figure 1:**
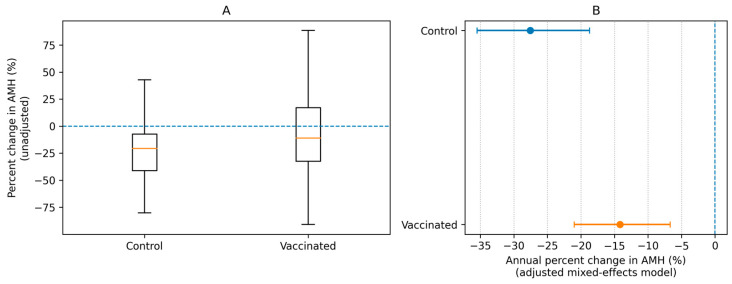
Unadjusted and adjusted AMH change by group. (**A**) Distribution of unadjusted percent change in AMH between baseline and follow-up measurements in the vaccinated and control groups (boxplots show median and interquartile range; whiskers indicate 1.5xIQR; outliers are not shown). The dashed horizontal line denoted no change (0%); (**B**) Adjusted annual percent change in AMH derived from the linear mixed-effects model of log-transformed AMH including fixed effects for time (years), group (vaccinated vs. control), and a time × group interaction, with additional adjustment for age, current smoking, gravidity, and parity, and a random intercept for participant. Points indicate model estimates and horizontal lines indicate 95% confidence intervals; the dashed vertical line denotes no annual change (0%).

In the adjusted mixed-effects model, AMH declined significantly over time in controls (time effect β = −0.322, 95% CI −0.438 to −0.207, *p* < 0.001), corresponding to an estimated −27.6% per year (95% CI −35.5% to −18.7%). The time × group interaction was positive and statistically significant (β = 0.170, 95% CI 0.027 to 0.312, *p* = 0.020), indicating a less steep decline in the vaccinated group. This corresponds to a slope ratio of 1.18 (95% CI 1.03–1.36) for vaccinated versus control participants. The implied annual change in the vaccinated group was −14.2% per year (95% CI −21.0% to −6.7%) (Table 3; Figure 1). Formal stratified analyses by reproductive age period (early/optimal/late) were not performed because subgrouping would substantially reduce sample sizes per stratum and yield imprecise estimates, particularly in the early subgroup (control *n* = 29).

**Figure 2:**
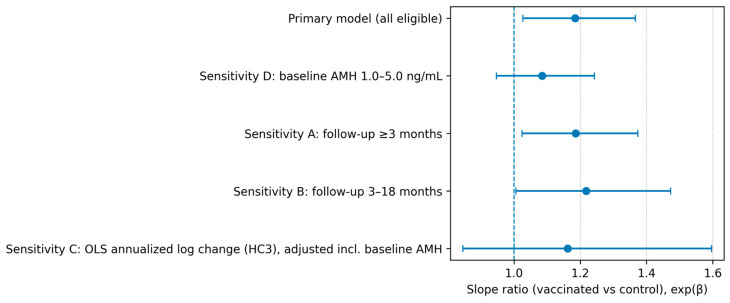
Sensitivity analyses of the group difference in annual AMH change. Points indicate slope ratios (vaccinated vs. control; exp(β)) and horizontal lines indicate 95% confidence intervals across the primary and sensitivity analyses (Table 4). The vertical reference line denotes no difference (slope ratio = 1).

Sensitivity analyses were summarized in Table 4. Results were consistent with the primary model when restricting follow-up to ≥3 months and to 3–18 months, with directionally consistent time × group effects and overlapping confidence intervals. In analyses restricted to participants with baseline AMH 1.0–5.0 ng/mL (vaccinated *n* = 82, control *n* = 67), the time × group interaction was attenuated and was not statistically significant (β = 0.082, *p* = 0.237; slope ratio 1.085, 95% CI 0.947–1.243). The estimated annual AMH decline in this restricted cohort was −25.3% (95% CI −32.2% to −17.7%) in controls and −18.9% (95% CI −26.3% to −10.7%) in vaccinated participants. Using an alternative OLS approach based on annualized log change adjusted for baseline AMH and covariates, the estimated rate ratio was directionally consistent but not statistically significant (1.162, 95% CI 0.846–1.596; *p* = 0.355) (Table 4; Figure 2). 

## 4. Discussion

In this retrospective cohort study with paired AMH measurements, we evaluated whether completion of a three-dose HPV vaccination series was associated with short-term changes in AMH compared with a contemporaneous control group. In adjusted mixed-effects modeling on the log scale, AMH declined over time in both groups; however, the rate of decline was less steep among vaccinated women than among controls (Table 3; Figure 1B). While the direction of the association suggested a modest difference in AMH alterations, the magnitude was small-to-moderate and should be interpreted cautiously in the context of measurement variability and residual confounding [18,22,23,24].

Our unadjusted analyses showed smaller apparent declines in AMH in the vaccinated group across multiple change metrics (absolute, percent, and log change) (Table 2; Figure 1A). Importantly, follow-up timing differed between groups, and baseline characteristics such as gravidity and smoking prevalence were not fully balanced (Table 1), underscoring the need for modeling approaches that account for time and the other confounders. After adjustment, the estimated annual decline in the control group was approximately −27.6% per year, whereas the vaccinated group showed an estimated −14.2% per year, with a statistically significant time × group interaction (Table 3; Figure 1B). These estimates reflect relative changes on the log scale and provide a standardized comparison across heterogeneous follow-up intervals.

The present findings are consistent with the broader body of evidence indicating no signal of impaired ovarian function or fertility outcomes following HPV vaccination. Large population-based studies have not demonstrated an increased risk of primary ovarian insufficiency after HPV vaccination [20], and epidemiologic analyses have not supported an association with infertility [25]. These observations are aligned with global post-licensure safety evaluations, including assessments by the WHO Global Advisory Committee on Vaccine Safety (GACVS), concluding that available evidence does not support an association between HPV vaccination and infertility or primary ovarian insufficiency [26]. Safety monitoring summaries and ongoing surveillance are also described by the U.S. Centers for Disease Control and Prevention (CDC) [27]. Collectively, these data support reassurance regarding ovarian safety concerns that may contribute to HPV vaccine hesitancy.

Notably, early safety concerns were amplified by case reports and small case series describing primary ovarian insufficiency (POI) or secondary amenorrhea temporally following HPV vaccination; however, such reports cannot establish causality because they lack an unexposed comparator, do not provide a denominator, and are vulnerable to temporal association and reporting/notoriety bias [19,28]. In addition, analyses based on passive surveillance systems (e.g., VAERS) are inherently limited by under-/over-reporting, variable diagnostic certainty, and stimulated reporting. In contrast, the most informative evidence comes from large population-based studies, which have not found an increased risk of POI after HPV vaccination, while acknowledging the rarity of the outcome and the consequent limits in detecting very small risk increases [20,29]. Accordingly, the overall body of evidence does not support a clinically meaningful adverse effect of HPV vaccination on ovarian function or fertility, although continued pharmacovigilance and well-designed longitudinal studies remain important.

From a biological standpoint, there is no established mechanism by which HPV vaccines would be expected to directly reduce follicular reserve. HPV vaccines are non-live, and their immunologic effects are not known to target ovarian tissue; major professional bodies and safety monitoring frameworks continue to support an overall favorable safety profile [26,27,30]. Therefore, if small between-group differences in AMH alterations are observed in routine-care datasets, they may plausibly reflect confounding by indication or healthcare utilization patterns (e.g., differential timing of laboratory testing, underlying gynecologic conditions prompting AMH measurement) rather than a direct vaccine effect. In addition, AMH is subject to both within-person biological variation and assay-related variability, which can contribute to apparent short-term fluctuations independent of true changes in ovarian reserve [18,22,23].

A key consideration is the clinical meaning of the observed differences. Although statistical significance was reached in the primary adjusted model (Table 3; Figure 1B), effect sizes in unadjusted comparisons were small to moderate (Table 2; Figure 1A), and the estimated between-group differences should be interpreted in light of AMH’s inherent variability [23,31] and the limitations of AMH as a standalone predictor of natural fecundity or future reproductive potential outside specific clinical contexts [24,32]. In this context, “statistically detectable” differences do not necessarily imply clinically meaningful changes in ovarian reserve or reproductive potential at the individual level.

Sensitivity analyses supported the general direction of the primary findings when restricting follow-up to ≥3 months and to 3–18 months, suggesting that extreme follow-up durations were unlikely to fully explain the main result (Table 4; Figure 2). However, when restricting the cohort to women with baseline AMH between 1.0 and 5.0 ng/mL, the time × group interaction was attenuated and no longer statistically significant (Table 4; Figure 2). This attenuation likely reflects reduced precision due to the smaller sample size after restriction (wider confidence intervals) and potentially greater homogeneity of baseline ovarian reserve within the restricted range. It also highlights that estimates may be influenced by baseline ovarian reserve distribution and residual confounding, and that conclusions should emphasize the absence of evidence for harm rather than implying a protective effect.

This study has limitations inherent to retrospective, record-based analyses. Vaccination and laboratory testing were not protocolized, and inclusion required paired AMH measurements, which may introduce selection bias and limit the feasibility of strict 1:1 matching between groups. Follow-up intervals also differed between groups because repeat AMH testing was ordered in routine care rather than at standardized time points; in vaccinated women, the second AMH measurement was often obtained in temporal proximity to completion of the three-dose series (reflecting real-world post-series follow-up), whereas in controls, repeat testing was driven by individualized clinical follow-up in general gynecology clinics. In our setting, patient-driven demand to “know ovarian reserve” has increased in recent years, and AMH is frequently requested in routine gynecology practice even in the absence of an immediate fertility intention; consequently, AMH testing may occur relatively independently of the presenting complaint, which may partially mitigate—but does not eliminate—selection differences inherent to retrospective sampling. The control cohort was assembled using a clinic-based sampling frame restricted to general gynecology outpatient clinics (defined by predefined clinic codes and/or a prespecified physician list), which standardizes the clinical context of AMH testing but may not fully represent the broader population of reproductive-age women. To reduce confounding by factors known to influence AMH, we excluded individuals with documented PCOS and endometriosis and those with recorded hormonal contraceptive use; however, incomplete documentation in routine records may lead to residual confounding. Age-period stratified analyses (early/optimal/late) were not performed because stratification would markedly reduce sample sizes per subgroup and yield underpowered, imprecise estimates, particularly in the early subgroup (control *n* = 29); thus, age-related effect modification cannot be ruled out. Body mass index was not routinely recorded, and its documentation was not missing at random (more often captured when clinicians perceived marked underweight/overweight habitus), precluding reliable adjustment for adiposity. Cycle day/phase at sampling had a variety in both cohorts; therefore, we did not analyze cycle-dependent hormones (FSH/estradiol) or operator-dependent AFC assessed by multiple clinicians, which may limit comparability with studies using standardized day-3 testing and centralized ultrasound. In addition, AMH assays and pre-analytical conditions may vary in routine practice, and inter-assay variability can affect the interpretation of small changes [18,22]. Strengths include the use of paired AMH measurements, explicit accounting for follow-up time, adjustment for key covariates, and multiple sensitivity analyses demonstrating generally consistent patterns (Table 4; Figure 2).

## 5. Conclusions

In this retrospective cohort with paired AMH measurements, HPV vaccination was not associated with evidence of clinically meaningful short-term impairment in ovarian reserve as assessed by AMH. Observed between-group differences in AMH alterations were modest and should be interpreted cautiously, given potential residual confounding and measurement variability. These findings provide additional reassurance for patients and clinicians regarding ovarian safety concerns related to HPV vaccination.

## Figures and Tables

**Table 3:** Primary adjusted analysis: mixed-effects model of log(AMH) over time.

Effect	Estimate (ln Scale)	95% CI (ln)	*p* Value	Estimate (Annual %)	95% CI (Annual %)
Annual change in controls (time)	−0.322	−0.438 to −0.207	<0.001	−27.6	−35.5 to −18.7
Vaccinated vs control difference in slope (time × group)	0.170	0.027 to 0.312	0.020	1.18	1.03 to 1.36
Annual change in vaccinated (time + time × group)	−0.152	−0.236 to −0.070	−	−14.2	−21.0 to −6.7

Linear mixed-effects model with random intercept for participant and fixed effects for time (years), group, time × group interaction, age, current smoking, gravidity, and parity. AMH was analyzed on natural log scale; annual % changes were derived as (e^β − 1) × 100. Slope ratio was e^(β_[time × group]).

**Table 4:** Sensitivity analyses of the group difference in annual AMH change.

Analysis	Vaccinated (*n*)	Control (*n*)	Time × Group β (ln/yr)	*p*	Slope Ratio e^β (95% CI)	Vaccinated Annual % Change (95% CI)	Control Annual % Change (95% CI)
**Primary model:** all eligible	158	106	0.170	0.020	1.185 (1.027 to 1.366)	−14.2 (−21.0 to −6.7)	−27.6 (−35.5 to −18.7)
**Sensitivity D:** baseline AMH 1.0–5.0 ng/mL	82	67	0.082	0.237	1.085 (0.947 to 1.243)	−18.9 (−26.3 to −10.7)	−25.3 (−32.2 to −17.7)
**Sensitivity A:** follow-up ≥3 months	140	105	0.170	0.023	1.186 (1.024 to 1.374)	−14.1 (−21.2 to −6.4)	−27.6 (−35.7 to −18.4)
**Sensitivity B:** follow-up 3–18 months	131	102	0.197	0.043	1.218 (1.006 to 1.473)	−13.9 (−25.2 to −0.9)	−29.3 (−37.8 to −19.6)
**Sensitivity C:** OLS annualized log change (HC3), adjusted incl. baseline AMH	158	106	0.150	0.355	1.162 (0.846 to 1.596)	—	—

The primary model is a linear mixed-effects model of log(AMH) with a random intercept and fixed effects for time (years), group, time × group, age, current smoking, gravidity, and parity. Sensitivity D has a baseline AMH of 1.0–5.0 ng/mL. Sensitivity A has a follow-up period of ≥3 months. Sensitivity B has a follow-up period of 3–18 months. Sensitivity C uses OLS on annualized log change with HC3 robust standard errors and adjusts for baseline log AMH (ln_pre) and covariates (age, current smoking, gravidity, and parity).

## Data Availability

The raw data supporting the conclusions of this article will be made available by the authors on request.
